# Curious Accident at the Paris Electrical Exhibition

**Published:** 1883-10-20

**Authors:** 


### CURIOUS ACCIDENT AT THE PARIS ELECTRICAL EXHIBITION.

The Scientific American quotes from a letter to the London Times, an account of an extraordinary occurrence at the great "Parisian Electric Exhibition." A gentleman was explaining a Brush Dynamo-Electric machine, when part of the conducting wire was not insulated and was lying on the floor. He touched the stand of a lamp which formed part of the conducting system. His body then formed a connection through the ground to the naked wire, and contracted his muscles so as to cause his hand to clinch the lamp. Ten lamps were in circuit at the time, and so much current was passed through him that eight of them were extinguished. He was powerless to unclasp his hand. Every muscle in his body was paralyzed. His face was distorted ; his lungs were so acted upon that he could scarcely breathe. He could only utter a faint and unnatural cry. The workmen in the place fled from the workshop, believing that some explosion was about to happen. A friend came up and tried to unlock his hand. It was impossible. He then lifted his legs from the ground. This broke the circuit and his hands were released, while burning sparks flew to his hands in the action of breaking the circuit. He was insensible, but has since then greatly recovered, and has devised an improvement to the lamp which will prevent a recurrence of such an accident.—*Pacific M. and S. Jour.*

